# Synergistic Sequential Emission of Fractional 10.600 and 1540 nm Lasers for Skin Resurfacing: An Ex Vivo Histological Evaluation

**DOI:** 10.3390/medicina58091308

**Published:** 2022-09-19

**Authors:** Steven Paul Nisticò, Luigi Bennardo, Tiziano Zingoni, Laura Pieri, Irene Fusco, Francesca Rossi, Giada Magni, Giovanni Cannarozzo

**Affiliations:** 1 Department of Health Sciences, Magna Graecia University, 88100 Catanzaro, Italy; 2 El.En. Group, 50041 Calenzano, Italy; 3 Lasers in Dermatology Unit, Department of Dermatology, University of Rome Tor Vergata, 00133 Rome, Italy; 4 Institute of Applied Physics “Nello Carrara”, National Research Council (IFAC-CNR), Sesto Fiorentino, 50019 Florence, Italy; 5 Unit of Lasers in Dermatology, University of Roma, Tor Vergata, 00133 Rome, Italy

**Keywords:** skin rejuvenation, fractional ablative and non-ablative lasers, synergic sequential emissions

## Abstract

*Background:* Fractional ablative and non-ablative lasers are useful treatments for skin rejuvenation. A procedure that provides the sequential application of fractional ablative followed by non-ablative laser treatment may reduce patients’ downtime and deliver better cosmetic results than with either laser alone. *Objective:* The purpose of the current study was to demonstrate the ameliorative and therapeutic effects in skin remodeling of the synergistic use of the two laser wavelengths (fractional ablative CO_2_ and non-ablative 1540 nm) with three different types of pulse shapes, S-Pulse (SP), D-Pulse (DP) and H-Pulse (HP), through which the CO_2_ laser can emit, performing an ex vivo histological evaluation. *Methods:* In this prospective study, ex vivo sheep inner thigh skin was chosen due to its similarity to human skin tissue, and a histological evaluation was performed. Three irradiation conditions, using all of the three CO_2_ pulse shapes (alone or averaged), were investigated: (1) 10.600 nm alone, the sequential irradiation of the two wavelengths in the same perfectly controlled energy pulses (DOT) for the entire scan area; ((2) 10.600 nm followed immediately by 1540 nm; and (3) 1540 nm followed immediately by 10.600 nm). *Results:* When comparing ablative to sequential irradiations, the synergy of the two wavelengths did not alter the typical ablative pulse shape of the 10.600 nm laser alone. With the same CO_2_ pulse shape, the lesion depth did not vary with the synergy of the two wavelengths, while thermal lesion width increased compared to CO_2_ alone. The ablation rate was achieved, while the total thermal lesion coverage in the scanning area of CO_2_ − 1540 lasers was greater than when using CO_2_ alone and then the other sequential irradiation. *Conclusions:* This study provides important preclinical data for new and early uses of the novel 10.600/1540 nm dual-wavelength non-ablative fractional laser. The synergy of the two wavelengths enhanced all the benefits already available when using CO_2_ laser systems both in terms of tone strengthening, thanks to a greater shrinking effect, and in terms of stimulation and collagen remodeling thanks to a greater volumetric thermal effect.

## 1. Introduction

Fractional photothermolysis is a laser technology that causes a molecular cascade involving changes in cytokines levels, heat shock proteins (HSPs) and matrix metalloproteinases (MMPs), from 2 days post-treatment up to 3 months, thus inducing tissue renewal and contraction with collagen remodeling [1]. This kind of laser system generates thousands of microablative zones (MAZs) and microthermal zones (MTZs) of injury, surrounded by islands of normal tissue; for this reason, healing is faster as the keratinocyte migration distance is shorter, reducing the risk of side effects [2,3,4].

A more marked effect of tissue remodeling and shrinkage is certainly achieved with continuous ablation (standard resurfacing). However, this method involves greater side effects (postoperative bacterial and viral infections, persistent erythema, skin changes, postinflammatory pigmentation and possible scars) and a certainly prolonged healing time [5,6].

The use of the fractional ablative CO_2_ laser alone can sometimes induce hyperpigmentation (PIH) and prolonged bleeding, in correlation with the quantity of used energy. These adverse post-treatment responses are associated with acute inflammatory responses to skin heat damage: during the inflammatory process, there is an increase in arachidonic acid metabolites that stimulate melanocytes to synthesize melanin [7,8].

An alternative solution is the CO_2_ laser simultaneous emission and bipolar radiofrequency (the extremities of the scanner are equipped with two bipolar electrodes that emit radiofrequency, affecting the entire tissue scanning area), which induces different biological effects on the tissue, such as epidermal coagulation for a dermal resurfacing and a denaturation effect to produce new collagen for deeper remodeling [6].

This minimally invasive technology that combines laser energy with a bipolar radiofrequency system provides a synergy of pulses without increasing the side effects and postoperative complications, such as atrophic scars, postinflammatory hyperpigmentation, bacterial infections or hypopigmentation, in comparison with standard ablative treatments or other non-ablative laser systems for the management of various skin and aesthetic conditions [9].

This methodology modulates the coagulative and ablative effects well, with reduced healing times and with the same amount of stimulation achieved using the CO_2_ laser alone [10].

**Figure 1:**
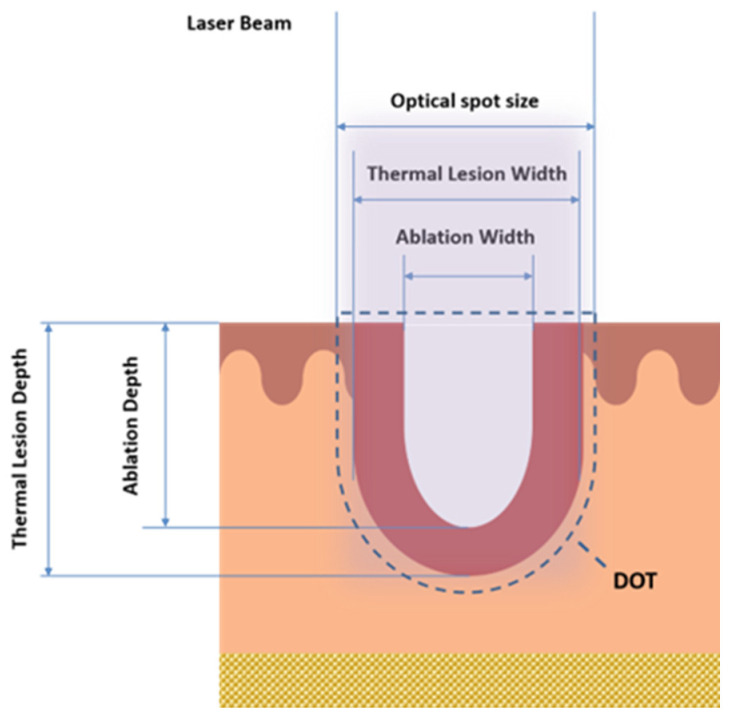
A schematic graphical representation of the DOT area (area that interests both MAZs and MTZs in the tissue), thermal Lesion Depth/Width and the Ablation Depth/Width.

In these scientific findings, it was observed that the non-ablative 1500–1600 nm lasers can also reach important depths in the reticular dermis of up to 2–3 mm, with a neocollagenesis effect. These wavelengths target water in the dermis, gently heating it to cause controlled thermal papillary dermis damage while providing epidermal protection, with consequent collagen remodeling, skin renewing and minimal side effects, retaining the downtime profile [11]. Histologic studies using the non-ablative laser showed neocollagenesis, epidermal thickening and increased elastic fibers [12]. Nevertheless, non-ablative lasers’ efficiency is lower than that of ablative ones, and they have been used for patients with moderate photoaging. Hence, there is a need to investigate the careful selection of wavelengths, with the special sequential emission on the single DOT (area that interests both MAZs and MTZs in the tissue, as shown in Figure 1 that synergistically enhances the therapeutic benefits (tissue coagulation and contraction effects), thanks to the reduced and optimized energy dose, and the increase in the safety profile with the reduction in post-treatment downtime.

The synergistic use of fractional non-ablative and ablative CO_2_ lasers demonstrates patients’ downtime and pain reduction during treatment sessions and better results with regard to skin rejuvenation than with either laser alone [13,14].

On this basis, the aim of the current study was to demonstrate the ameliorative and therapeutic effects for skin remodeling of the synergistic use of the two laser wavelengths (fractional ablative CO_2_ and non-ablative 1540 nm) with three different types of pulse shapes, S-Pulse (SP), D-Pulse (DP) and H-Pulse (HP), through which a CO_2_ laser can emit, performing an ex vivo histological evaluation.

## 2. Materials and Methods

Ex vivo sheep inner thigh skin was chosen due to its similarity to human skin tissue. The DuoGlide system (DEKA Mela Srl, Florence, Italy) used for this study is a multi-technology that incorporates 10.600 nm carbon dioxide (CO_2_) laser device (60 W) and 1540 nm diode laser (10 W), which can be used with the fractioned scanning units (μScan DOT). This scanner can deliver one or both wavelengths (1540 nm and 10.600 nm) in a sequential emission mode on the same DOT; this allows for a tunable balance between ablation and coagulation depths and new and more efficient treatments.

The system emits perfectly controlled energy/DOT by managing the power and the pulse duration (dwell time) parameters and, finally, the “DOT spacing” can be selected, which determines the area of the tissue not involved by CO_2_ irradiation.

The optical diameter of the CO_2_ spot is about 250 μm, and this technology also offers different CO_2_ pulse shapes, which are important in guaranteeing both the superficial ablation of the epidermis and the release of heat deeper into the dermis [9].

**Figure 2:**
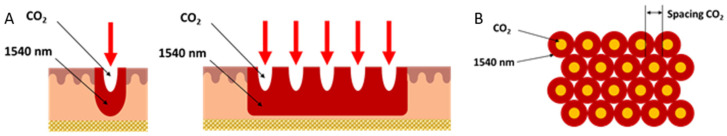
A schematic graphic representation of the action of the two wavelengths in order to better show the ablation and thermal effect of both laser emissions, alone or in combination. Front view (**A**) and top view (**B**).

The second wavelength at 1540 nm conveyed through the new miniaturized scanning systems allows a homogeneous, contiguous and non-coagulative heating of the entire scanning area to be achieved, reaching new and high dermal depths (not easily reachable with the ablative laser alone), thanks to spots of the order of 1000 µm emitted on the same axis as the DOT and thanks to the use of typical CO_2_ spacing parameters (approx. 500 μm) used in the literature for dermatological applications [9,11]. The sequential action with the CO_2_ and infrared wavelengths extends and enhances the thermal effect for a more effective treatment in tissue remodeling, always guaranteeing the healing times of the fractionated emission modes (Figure 2).

Three types of irradiations over an area of 15 × 15 mm^2^ were investigated: the effect of 10.600 nm alone, and in combination with 1540 nm for the same DOT of the entire scan area, specifically in this order: 10.600 nm followed immediately by 1540 nm and 1540 nm followed immediately by 10.600 nm.

**Figure 3:**
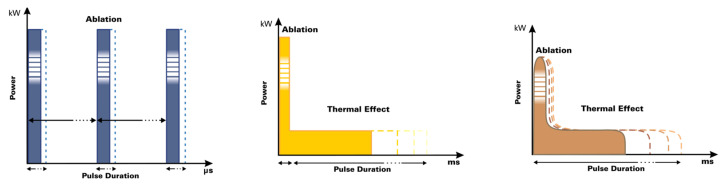
Schematic representation of the three pulse shapes (HP, DP and SP, respectively). Courtesy of DEKA MELA.

For each type of irradiation, three different pulse types of CO_2_ were used: S-Pulse (SP), D-Pulse (DP) and H-Pulse (HP) (see Figure 3).

The CO_2_ energy/DOT was set to about 44 mJ (~90 J/cm^2^@DOT) according to a clinical trial found in the literature [9,15], while the CO_2_ spacing was set to 500 µm. For the 1540 nm wavelength, 30 mJ (power 6 W and dwell time 5 ms) was used.

A total of 10 skin samples were used for the experiments.

Before laser exposure, the sheep skin was shaved, and skin samples of 2 cm × 2 cm were obtained. After laser exposure, biopsy specimens were immediately plunged in 4% formaldehyde (Bio-optica, Milano spa, Italy) solution for 5 days and then processed (dehydration, paraffin embedding and sectioning) for light microscopy. Sections were stained with hematoxylin and eosin and finally examined under an optical microscope (Eclipse 80i Microscope, NIKON, Shinagawa, Tokyo, Japan,) for the following measurements for each energy/DOT: coagulative thermal damage (Thermal Lesion Depth/Width (μm)) and ablation damage (Ablation Depth/Width (μm)) (Figure 3); in addition, the coagulation and ablation volumes were calculated according to (Table 1).

Finally, a shrinkage test was performed: the square skin samples (area: 4 cm^2^) were placed without tension on a smooth flat surface on top of a sheet of graph paper, and a 15 × 15 mm^2^ square was drawn on the sample; gel was placed between the samples and the graph paper in order to facilitate the possible shrinkage of the samples and to allow us to measure it. Samples were then irradiated: one with only 10.600 nm (TEST 1), one with only 1540 nm (TEST 2), and the other two with the sequential irradiation of the two wavelengths (10.600 nm followed immediately by 1540 nm (TEST 3) and 1540 nm followed immediately by 10.600 nm (TEST 4)). After irradiation, it was possible to measure the square drawn, thanks to the graph paper, and the tissue contraction through the formula: variation% = 100*(initial area − final area)/initial area. Each test was repeated 5 times, and the mean and standard deviation were calculated.

## 3. Results

**Figure 4:**
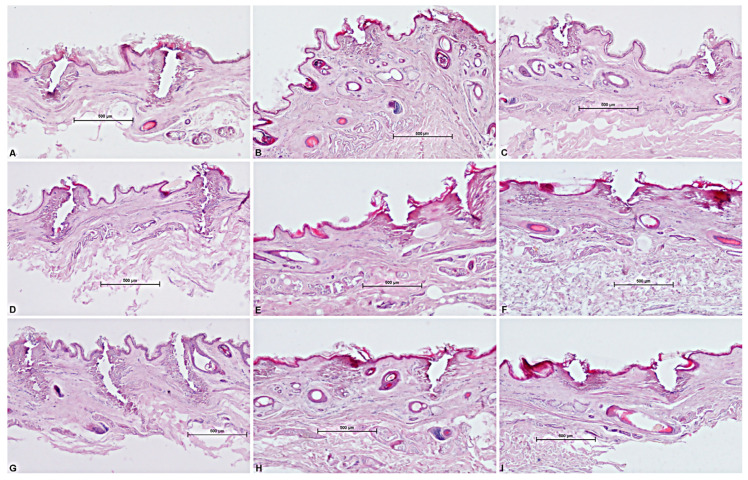
Histological outcomes in ex vivo sheep skin: top panel: only CO_2_ emission; middle panel: 10.600 nm and 1540 nm emissions and bottom: panel 1540 nm and 10.600 nm emissions. (**A**,**D**,**G**) H-Pulse; (**B**,**E**,**H**) S-Pulse; (**C**,**F**,**I**); D-Pulse.

An analysis of the samples treated with the 10.600 nm laser shows that the three pulse shapes (SP, DP and HP) allowed us to obtain different patterns of spatial/lateral damage heat distribution, tissue shrinkage profiles and ablation area shapes (Figure 4).

This technology, with a range of available pulse modes, allowed the induction of different tissue biological effects, stimulating new collagen production in the dermis, naturally regenerating the tissue structure and improving skin tightening or softness. In detail, the S-Pulse mode was able to induce a homogeneous coagulation of the surrounding tissues by acting with a more circular form of ablation, while the D-pulse mode generated a greater contraction of the ablation columns and a more circumscribed coagulation by acting in a more incisive way on the reticular dermis. Following the D-Pulse ablation, the histological analysis showed a depression of the epidermis portion not involved in the phenomenon of hardening and caused by the immediate heat wave. This was attributed to the deeper action exerted by this modality, which also involves the reticular dermis and induces a greater shrinkage even in the vertical direction.

Thanks to the higher peak power of HP, compared to the SP and DP modes described above, this mode also makes it possible to obtain additional effects on the tissues, such as a greater ablation, than the other emission modes applied.

**Figure 5:**
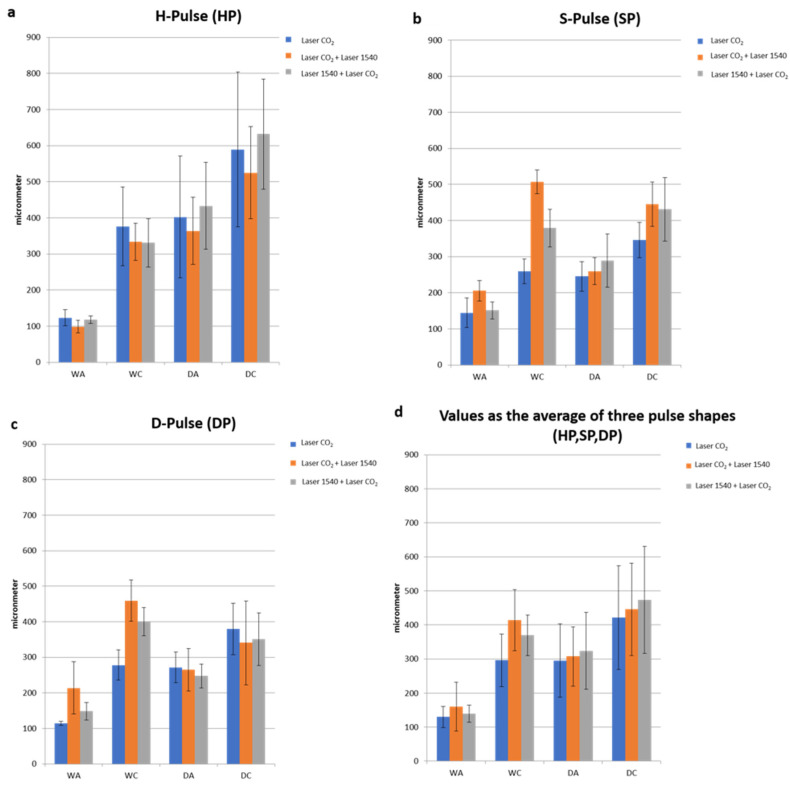
Graphic representation of the values of ablation width (WA), thermal lesion width (WC), ablation depth (DA) and thermal lesion depth (DC) for the three laser irradiation emissions. Values are calculated for each single pulse shape (**a**–**c**) and as the average of the three pulse shapes (**d**).

**Figure 6:**
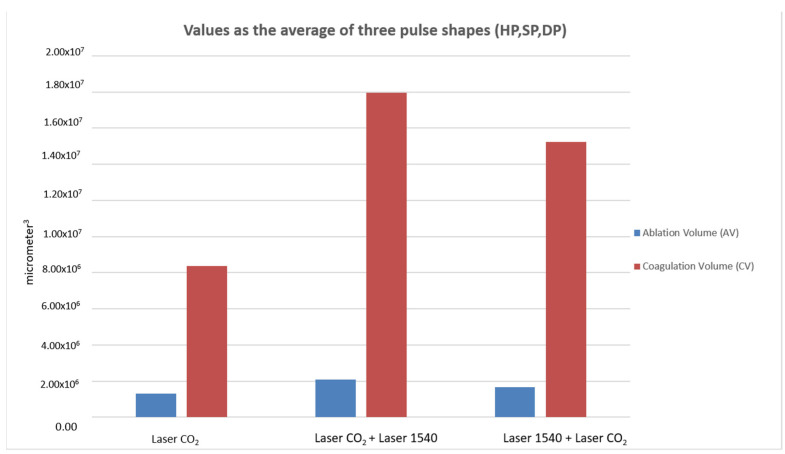
Graphic representation of ablation volume (AV) and coagulation volume (CV) for the three types of laser emissions calculated based on the average of the CO_2_ pulse shapes (HP, SP, DP).

When comparing ablative to sequential irradiations, the synergy of the two wavelengths did not alter the typical pulse shape (HP-SP-DP) of the 10.600 nm laser alone. With the same pulse shape, the DA and the WA did not vary with the synergy of the two wavelengths, while WC (coagulation) increased compared to CO_2_ alone and determined the increase in coagulation volume for the synergy of the two wavelengths (10.600 nm and 1540 nm) compared to CO_2_ alone (Figure 4, Figure 5 and Figure 6).

A significant variation (*p* < 0.01) in WC values was observed (Table 2) for SP and DP, as they already had their own thermal properties without the addition of the 1540 nm wavelength, which were amplified by CO_2_.

Thus, the second wavelength did not increase the WC of the HP impulse, which was already cold in the CO_2_ emission mode.

In all cases of wrinkles and laxity, the usage of the CO_2_ + 1540 emission led to a rise in the lateral shrinkage effect as needed without increasing the ablative effect.

The total ablation coverage in the scanning area did not change, so the same ablation rate was achieved for all three types of irradiation (the total ablation coverage was about 2% of the 15 × 15 mm scan area); instead, the total thermal lesion coverage in the scanning area of 10.600 nm and 1540 nm wavelengths was greater than CO_2_ alone and the following sequential irradiation (1540 nm and 10.600 nm). An increase in the coagulation zone caused a greater shrinkage of the tissue, but still fell within a range that does not affect tissue healing. The 10.600–1540 nm sequence enhanced the effects, both in terms of strengthening of tone, thanks to a greater shrinkage effect, and in terms of stimulation thanks to a greater volumetric thermal effect.

**Figure 7:**
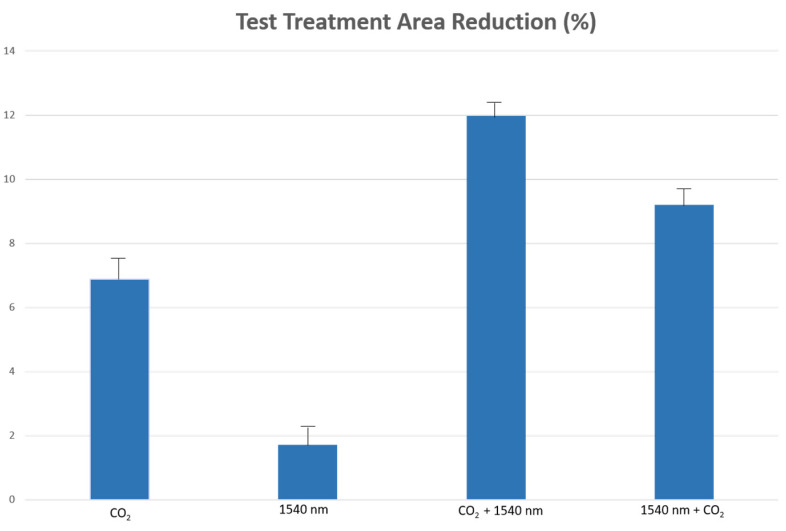
Percentage of reduction in the treatment area with different wavelengths and combinations of them. The combined use of the two wavelengths demonstrated a greater contraction effect on ex vivo skin.

Moreover, the combined use of the two wavelengths demonstrated a greater contraction effect on ex vivo skin. In particular, the CO_2_ − 1540 nm sequential emission mode (TEST 3) led to an area reduction of 11.96%, much higher than the reduction in areas obtained with the single wavelengths (TEST 1). Even the percentage values of the reduction in areas from the synergy of the two wavelengths (area reduction CO_2_ − 1540 nm: 11.9 ± 0.5% (TEST 3); area reduction 1540 nm − CO_2_: 8.9 ± 0.6% (TEST 4)) were greater than the sum of the percentage values obtained from the use of the single wavelengths (area reduction CO_2_: 6.8 ± 0.8% (TEST 1), area reduction 1540 nm: 1.8 ± 0.5% (TEST 2) (Figure 7).

## 4. Discussion

In this study, coagulative thermal damage and ablation damage were evaluated following the use of a 10.600 nm wavelength alone and the combined use of 10.600 nm and 1540 nm and vice versa.

Ablative fractional lasers have faster results, but are often associated with the highest complication rate, with greater invasiveness, loss of skin integrity and longer recovery time and downtime [6].

Non-ablative fractional lasers, on the other hand, are associated with a low rate of adverse effects and are applicable to almost any patient, but repeated treatments may be required to achieve the wanted results [12,16,17,18,19].

The preclinical histological analysis by Tenna et al. [9] shows that the two pulse forms, S-Pulse and D-Pulse, allow for different models of spatial heat distribution, shapes of the ablation area, distribution of lateral thermal damage and fabric shrinkage profiles to be obtained.

Specifically, the S-Pulse mode, working more selectively, creating a circular U-shaped ablation shape, induces a homogeneous coagulation of the surrounding tissues, suggesting the validity of its clinical application in the treatment of atrophic scars.

The D-Pulse mode induces greater shrinkage of the ablation columns such as a V-shape and more circumscribed coagulation, thus improving skin elasticity and texture [9].

Furthermore, the pulsed shapes combined with bipolar radiofrequency enhance the effects of the CO_2_ laser treatment by deeply reshaping the tissues, toning the flaccidity and stimulating the activity of fibroblasts for the production of new collagen.

Actually, in the literature, the best results are often achieved by combining treatments (radiofrequency, cosmetics and laser), resulting in a reduction in side effects, and favoring the restoration of the correct barrier function.

The combined use of radiofrequency and fractional CO_2_ resurfacing lasers allows lower energies to be used for both energy forms; it seems to intensify the thermal effects on the treated tissues, producing better results in less time and with fewer sessions without increasing the risks or side effects related only to the treatment with the CO_2_ laser or radiofrequency; moreover, the synergy of the CO_2_ laser and radiofrequency created different biologic effects, which range from dermal stimulation for new collagen production to regeneration of tissue. [6,8,9,10].

With a wavelength of 1540 nm, Mordon and colleagues [20] proved in an animal model that this laser could cause the synthesis of neocollagen and the strengthening of collagen without damaging the overlying epidermis; therefore, 1540 nm induces the same effects at the tissue level as compared to radiofrequency, but with three important advantages that certainly improve its operability: greater thermal gradient, shorter emission time (milliseconds vs. seconds) and laser selectivity.

**Figure 8:**
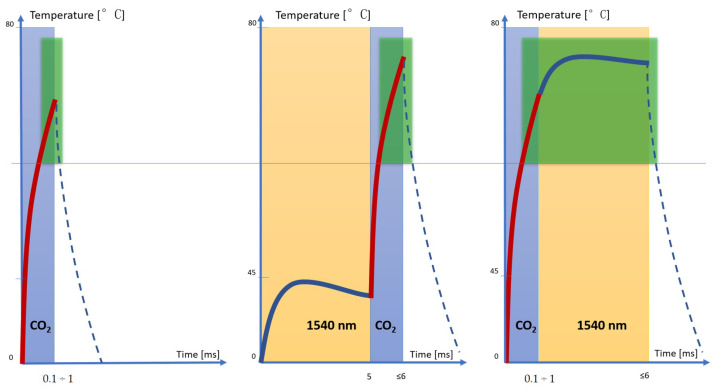
Qualitative graphic representation of denaturation volume of the different emissions interactions with tissue. (Temperature (°C) range: 0–80; time (ms) range: 0–5). The 10.600–1540 nm sequence emission led to a greater coagulation effect and, therefore, greater shrinkage of the tissue.

The emission of the CO_2_ laser allows the necessary temperatures for denaturing the collagen fibers to be reached in a few milliseconds, but these temperatures drop rapidly, allowing only the area surrounding the ablative vallus to be coagulated. When using both wavelengths in the 1540–10.600 nm sequence, the 1540 nmwavelength preheats the tissue and allows the CO_2_ to more easily reach the target temperature and hold it for a slightly longer time than in the previous case, thus allowing the volume of coagulation in the tissue to increase. In the sequence 10.600–1540 nm, on the other hand, CO_2_ alone allowed us to reach the temperature of denaturation of the collagen fibers (which ranges between 60 °C and 80 °C ), as demonstrated by the morphological aspect of the histologies, and 1540 allowed us to maintain it for a longer time (about few seconds) than the two previous cases. For this reason, the 10.600–1540 nm sequence emission allowed us to obtain a greater coagulation effect and, therefore, greater shrinkage of the tissue (Figure 8).

Moreover, with the 1540 nm wavelength alone, it was possible to selectively reach depths of 2.5 mm with controlled temperature rises between 40 and 45 °C (data not shown). These temperatures of reversible action have important biological effects of biostimulation [11,16]. The fundamental principle on which the tissue biostimulation process is based can be easily understood because it follows the classic wound healing process characterized by the following phases: inflammation, proliferation and remodeling. The tissue response to heat shock induces rapid and transient changes in cellular metabolism, due to photodissociation of inhibitory nitric oxide from cytochrome c oxidase, resulting in increased production of ATP and cell activity, leading to the production of HSPs, matrix MMPs, interleukins (ILs) and transforming growth factor betas (TGF-bs) [17,18,19,20,21].

In the literature, it is well-described that the new collagen production is controlled not only by the collagen injury with subsequent repair, but also by inflammatory mechanisms that lead to the production of HSPs; HSPs induce TGF-beta, which subsequently promotes the chemotaxis of fibroblasts, resulting in procollagen formation. As a result, MMP levels rise, contributing to matrix remodeling via fibrillar collagen cleavage. In fact, it is clearly demonstrated that temperatures below 45 °C have not been able to induce histological changes in the tissue, but only repeated temperatures above 48 °C can induce histological damage to the epidermis. Later, the proliferation phase is characterized by the migration of fibroblasts and the development of new vessels to supply nutrients and oxygen to repair damaged cells. Finally, the last remodeling phase occurs with the continuous deposition of well-organized new collagen bundles [22,23].

Therefore, the second wavelength, 1540 nm alone, does not introduce any effect that can morphologically modify the tissue, but instead promotes neocollagenesis while completely sparing the epidermis from damage, and when 1540 nm is used in combination with CO_2_, its coagulative peculiarities are increased and enhanced, thus splitting the proportionality between ablation and coagulation. This coagulative extension effect under the healthy epidermis, i.e., between the two consecutive CO_2_ DOTs, generates, thanks to 1540 nm, a more uniform remodeling and simulates the request that was obtained with traditional resurfacing but now has healing times equivalent to fractional CO_2_ alone.

The higher coagulation rate related to the single DOT is subsequently mitigated within the fractional scan in terms of safety, and this minimally invasive technology guarantees long-lasting improvement.

Our results agree with a study by Mezzana [13] conducted on 20 patients and with the study by Snast and collaborators [24], who affirm that the combined use of two wavelengths, 10.600 nm and near infrared, does not modify the ablation of CO_2_ laser, but increases the coagulation zone, improving the effectiveness and safety of the treatment and reducing downtime.

The synergy of the two wavelengths enhances all the benefits already available when using CO_2_ laser systems and supports a new CO_2_ − 1540 nm sequential emission mode within each single DOT. The user, depending on the specific case, can proceed using only the CO_2_ wavelength with the benefits of over thirty years of fractional experience or the CO_2_ − 1540 nm or 1540 nm − CO_2_ sequences in order to enhance the effects both in terms of strengthening of tone, thanks to a greater shrinkage effect (for example, wrinkles and laxity), and in terms of stimulation thanks to a greater volumetric thermal effect (for example in the remodeling of atrophic scars), respectively. The device, by combining laser energies, is able to guarantee a significant reduction in postoperative complications and side effects compared to other non-ablative laser systems in use for various dermatological/aesthetic conditions and standard ablative treatments [13].

## 5. Conclusions

With the variety of available pulse modes, this new technology allows the induction of various tissue biological effects, such as stimulating new collagen production in the dermis, naturally regenerating tissue structure and improving skin tightening or softness. Histological examination shows that the higher peak power of HP, leads to additional effects on the tissues, such as a greater ablation, compared to the other emission modes applied. In the sequence 10.600–1540 nm, CO_2_ alone allows us to reach the temperature of denaturation of the collagen fiber as demonstrated by the morphological aspect of the histologies, and 1540 allows us to maintain it for a longer time. As a result, the 10.600 nm-1540 nm sequence emission allows us to achieve a greater coagulation effect and, as a result, greater tissue shrinkage. Finally, the combined use of the two wavelengths demonstrated a greater contraction effect on ex vivo skin, as clearly demonstrated by the test treatment area reduction.

## Figures and Tables

**Table 1:** Parameter abbreviations and coagulation/ablation volume calculations.

Thermal Lesion Width	(WC)
Thermal Lesion Depth	(DC)
Ablation Width	(WA)
Ablation Depth	(DA)
Ablation Volume (AV)	π*(WA/2)^2^*DA/3
Coagulation Volume (CV)	[π*(WC/2)^2^*DC/3]-AV

**Table 2:** *p*-values and Mean ± DevST of WA, WC, DA and DC for each pulse shape for the three laser irradiation emissions.

**H-Pulse (*n* = 5)**	**WA** *[Mean* ± *DevST]*	**WC** *[Mean* ± *DevST]*	**DA** *[Mean* ± *DevST]*	**DC** *[Mean* ± *DevST]*
Laser CO_2_	123 ± 22 μm	376 ± 109 μm	402 ± 169 μm	590 ± 215 μm
Laser CO_2_ + Laser 1540	98 ± 18 μm	334 ± 51 μm	364 ± 93 μm	525 ± 128 μm
Laser 1540 + Laser CO_2_	118 ± 11 μm	331 ± 67 μm	433 ± 121 μm	632 ± 153 μm
“CO_2_” vs “CO_2_ + 1540”	-	-	-	-
“CO_2_” vs “1540 + CO_2_”	-	-	-	-
**S-Pulse** (*n* = 5)	**WA** *[Mean* ± *DevST]*	**WC** *[Mean* ± *DevST]*	**DA** *[Mean* ± *DevST]*	**DC** *[Mean* ± *DevST]*
Laser CO_2_	144 ± 41 μm	259 ± 34 μm	245 ± 40 μm	346 ± 49 μm
Laser CO_2_ + Laser 1540	206 ± 29 μm	507 ± 33 μm	260 ± 37 μm	445 ± 61 μm
Laser 1540 + Laser CO_2_	151 ± 23 μm	379 ± 52 μm	289 ± 74 μm	432 ± 88 μm
“CO_2_” vs “CO_2_ + 1540”	-	*p* < 0.01	-	-
“CO_2_” vs “1540 + CO_2_”	-	*p* < 0.01	-	-
**D-Pulse (*n* = 5)**	**WA** *[Mean* ± *DevST]*	**WC** *[Mean* ± *DevST]*	**DA** *[Mean* ± *DevST]*	**DC** *[Mean* ± *DevST]*
Laser CO_2_	114 ± 5 μm	278 ± 42 μm	272 ± 43 μm	379 ± 73 μm
Laser CO_2_ + Laser 1540	214 ± 73 μm	460 ± 59 μm	265 ± 60 μm	341 ± 118 μm
Laser 1540 + Laser CO_2_	149 ± 25 μm	400 ± 40 μm	248 ± 34 μm	351 ± 74 μm
“CO_2_” vs “CO_2_ + 1540”	-	*p* < 0.01	-	-
“CO_2_” vs “1540 + CO_2_”	-	*p* < 0.01	-	-

## Data Availability

Data supporting this study findings are available on request from the corresponding author (IF).
